# Multi-model order spatially constrained ICA reveals highly replicable group differences and consistent predictive results from resting data: A large N fMRI schizophrenia study

**DOI:** 10.1016/j.nicl.2023.103434

**Published:** 2023-05-17

**Authors:** Xing Meng, Armin Iraji, Zening Fu, Peter Kochunov, Aysenil Belger, Judy M. Ford, Sara McEwen, Daniel H. Mathalon, Bryon A. Mueller, Godfrey Pearlson, Steven G. Potkin, Adrian Preda, Jessica Turner, Theo G.M. van Erp, Jing Sui, Vince D. Calhoun

**Affiliations:** a Tri-Institutional Center for Translational Research in Neuroimaging and Data Science (TReNDS), Georgia State, Georgia Tech, Emory University, Atlanta, GA, USA; b Brainnetome Center and National Laboratory of Pattern Recognition, Institute of Automation, Chinese Academy of Sciences, Beijing, China; c University of Chinese Academy of Sciences, Beijing, China; d Maryland Psychiatric Research Center, Department of Psychiatry, School of Medicine, University of Maryland, Baltimore, MD, USA; e Department of Psychiatry, University of North Carolina, Chapel Hill, NC, USA; f Department of Psychiatry, University of California San Francisco, San Francisco, CA, USA; g San Francisco VA Medical Center, San Francisco, CA, USA; h Department of Psychiatry and Biobehavioral Sciences, University of California Los Angeles, Los Angeles, CA, USA; i Department of Psychiatry, University of Minnesota, Minneapolis, MN, USA; j Departments of Psychiatry and Neuroscience, Yale University, School of Medicine, New Haven, CT, USA; k Department of Psychiatry and Human Behavior, University of California Irvine, Irvine, CA, USA; l Department of Psychology, Georgia State University, Atlanta, GA, USA; m Clinical Translational Neuroscience Laboratory, Department of Psychiatry and Human Behavior, University of California Irvine, Irvine, CA, USA

**Keywords:** Functional network connectivity(FNC), Component number, Spatially constrained ICA, Resting fMRI, Machine learning, Intrinsic connectivity networks

## Abstract

Brain functional networks identified from resting functional magnetic resonance imaging (fMRI) data have the potential to reveal biomarkers for brain disorders, but studies of complex mental illnesses such as schizophrenia (SZ) often yield mixed results across replication studies. This is likely due in part to the complexity of the disorder, the short data acquisition time, and the limited ability of the approaches for brain imaging data mining. Therefore, the use of analytic approaches which can both capture individual variability while offering comparability across analyses is highly preferred. Fully blind data-driven approaches such as independent component analysis (ICA) are hard to compare across studies, and approaches that use fixed atlas-based regions can have limited sensitivity to individual sensitivity. By contrast, spatially constrained ICA (scICA) provides a hybrid, fully automated solution that can incorporate spatial network priors while also adapting to new subjects. However, scICA has thus far only been used with a single spatial scale (ICA dimensionality, i.e., ICA model order). In this work, we present an approach using multi-objective optimization scICA with reference algorithm (MOO-ICAR) to extract subject-specific intrinsic connectivity networks (ICNs) from fMRI data at multiple spatial scales, which also enables us to study interactions across spatial scales. We evaluate this approach using a large N (N > 1,600) study of schizophrenia divided into separate validation and replication sets. A multi-scale ICN template was estimated and labeled, then used as input into scICA which was computed on an individual subject level. We then performed a subsequent analysis of multiscale functional network connectivity (msFNC) to evaluate the patient data, including group differences and classification. Results showed highly consistent group differences in msFNC in regions including cerebellum, thalamus, and motor/auditory networks. Importantly, multiple msFNC pairs linking different spatial scales were implicated. The classification model built on the msFNC features obtained up to 85% F1 score, 83% precision, and 88% recall, indicating the strength of the proposed framework in detecting group differences between schizophrenia and the control group. Finally, we evaluated the relationship of the identified patterns to positive symptoms and found consistent results across datasets. The results verified the robustness of our framework in evaluating brain functional connectivity of schizophrenia at multiple spatial scales, implicated consistent and replicable brain networks, and highlighted a promising approach for leveraging resting fMRI data for brain biomarker development.

## Highlights

• Multi-model order spatially constrained ICA reveals highly replicable group differences and consistent predictive results from resting data of schizophrenia.
• Multi-model order ICA provides a comprehensive way to study brain functional network connectivity within and between multiple spatial scales, highlighting findings that would have been ignored in a single model order analysis.
• Cross-model order ICA provides useful information and is widely applicable to other areas since it allows us to capture coupling between smaller and larger networks.
• Cross-model order ICA study improves classification accuracy in detecting schizophrenia.
• Our framework was built and tested leveraging a large N (N > 1,600) dataset, and revealed highly replicable group differences and consistent predictive results across datasets.

## 1 Introduction

### 1.1 Background

Schizophrenia is a severe psychiatric disease characterized by hallucinations, delusions, loss of initiative, and cognitive dysfunction (Van Den Heuvel & Fornito, 2014), that typically emerges in late adolescence or early adulthood (Sadeghi et al., 2022). Schizophrenia is a heterogeneous syndromic diagnosis of exclusion, that lacks unique symptoms, and coordination between thoughts, actions, and emotions (Shoeibi et al., 2021). Schizophrenia is diagnosed clinically by both positive and negative symptoms (American Psychiatric Association (2013)). Schizophrenia has been hypothesized as a developmental disorder of disrupted brain function, which can be characterized by functional dysconnectivity and changes in functional integration (Friston and Frith, 1995, Stephan et al., 2006). Studying functional connectivity can provide important information about brain functional integration and its schizophrenia changes, potentially improving our understanding of the actual brain pathology, thus eventually improving treatments and care for individuals with schizophrenia (Iraji et al., 2021a, Iraji et al., 2021b).

Group ICA (Calhoun et al., 2001a, Calhoun et al., 2001b) has been widely applied for investigating functional network biomarkers in neuroimaging data. Group ICA is typically used to extract brain functional networks from multiple subjects in a fully data-driven manner. However, comparison of these networks across datasets can be challenging if they are analyzed separately because the components will be randomly sorted at each run. While one can use regression approaches to estimate the networks in new subjects, this can decrease efficiency as regression neither ensure maximal independence within each subject nor does it fully adapt the spatial networks to the new data (Erhardt et al., 2011). Consequently, when leveraging higher-order statistics, previous work has shown the statistical and classification performance of regression-based approaches is reduced compared to ICA solutions (Salman et al., 2019).

In contrast, spatially constrained ICA (scICA) of fMRI (Lin et al., 2010, Du and Fan, 2013) was proposed to overcome difficulties in identifying components of interest and determining the optimal number of components in ICA analysis because scICA incorporates available spatial prior information about the sources into standard blind ICA. Previous studies (Sui et al., 2009, Qi et al., 2018, Salman et al., 2019) have demonstrated the benefit of using scICA in functional MRI analysis. Recent work has proposed deriving component priors by identifying replicable component maps across multiple large datasets analyzed separately, then using a template in scICA. scICA has been used in a variety of studies to date (Du et al., 2019, Du et al., 2020), and this approach has multiple strengths: 1) fully automated, avoiding the need to select, label, and order components, 2) adapts the component maps and timecourses to individual subjects, making cross-validation easier, and 3) provides comparability across studies. A recent scICA approach called MOO-ICAR (Du et al., 2020) was implemented using the GIFT software toolbox (https://trendscenter.org/software/gift) (Iraji *et al.,* 2020). The MOO-ICAR approach utilized components from the Neuromark_fMRI_1.0 template (available in GIFT version 4e) as the references to extract subject-specific independent component maps and their time courses. The template includes fifty-three ICNs and is arranged into seven functional domains using visual inspection and atlas labels based on the peak coordinates for each component, including 2 auditory, 4 cerebellar, 17 cognitive control, 7 default mode, 5 subcortical, 9 somatomotor, and 9 visual components. After obtaining the subject-specific time-courses, the ICN time courses were linearly detrended and filtered between 0.01 and 0.15 Hz prior to FNC calculations. The Pearson correlation coefficient was then calculated between time courses of ICNs and r-to-z transformed, resulting in a 53 * 53 FNC matrix for each participant (DeRamus et al., 2022). The MOO-ICAR framework (Du et al., 2019) estimates subject-specific ICs that provide more optimal independence and better spatial correspondence across different subjects and achieve higher spatial and temporal accuracy compared to existing ICA methods. The MOO-ICAR approach has been applied in multiple studies. For example, researchers have used the Neuromark IC template to estimate subject-specific networks that were then used as features in a support vector machine (SVM)-based framework (Osuch et al., 2018, Du et al., 2019, Du et al., 2020) to predict response to medication (either antidepressants or mood stabilizers) in bipolar disorder and major depressive disorder patients.

Most previous research has focused on priors derived from a single ICA model order (i.e., 53 networks from 100 components estimated), ignoring the importance of capturing functional information at different levels of spatial granularity as well as the between-order information. In recent work, we have shown the advantage of working with multiple spatial scales (i.e., multi-model order ICA) (Iraji et al., 2021b, Meng et al., 2021). Multi-model order ICA provides a comprehensive way to study brain functional network connectivity within and between multiple spatial scales, highlighting findings that would have been ignored in a single model order analysis. Previous studies (Iraji et al., 2021a, Meng et al., 2021) have highlighted the benefit of studying functional network connectivity in schizophrenia using multiple spatial scales.

Here we propose the use of ICNs resulting from scICA with multiple spatial scales to fill this gap and also apply this approach to a large N study including validation on an independent dataset. Our hypothesis was that combining scICA and msFNC would yield group differences and classification results that are robust to data collected at different sites and with different demographics. Combining scICA and msFNC to detect group differences in schizophrenia allows us to leverage the known benefits of both approaches. To the best of our knowledge, no studies have shown the robustness of scICA in the context of capturing group differences at single or multiple spatial scales. To verify our hypothesis, in this study, we proposed a framework to evaluate multi-model order scICA using MOO-ICAR to capture robust, replicable schizophrenia-related alterations and identify potential biomarkers for schizophrenia classification. We used a recently developed multi-model order ICA template (Meng et al., 2021) and together with the proposed scICA based framework addressed in this work to identify consistent predictive ICNs in schizophrenia. To evaluate the robustness of our framework, we built our framework on one dataset and evaluated the performance on an independent dataset.

## 2 Literature review

### 2.1 Multi-model order ICA / multiple spatial scales

The brain can be segregated into distinct functional sources (e.g., ICNs), which dynamically interact with each other (i.e., functional integration), and brain function has been modeled as coordination and interaction between functional sources, which has been studied using the principles of segregation and integration (Genon et al., 2018). ICN is a temporally synchronized pattern of the brain, a good estimation of a functional source. The ICN time course describes its functional activity over time, while its spatial pattern indicates the contribution of spatial locations to ICN. The spatial scale of ICNs can be set effectively using the model orders of ICA. Thus, we can study brain segregation and estimate ICNs at different spatial scales by using ICA with different model orders (Iraji et al., 2022a, Iraji et al., 2022b). Low model order ICA results in large-scale spatially distributed ICNs (Damoiseaux et al., 2008, Iraji et al., 2016), while high model order results in more spatially granular ICNs (Iraji et al., 2009, Iraji et al., 2019, Allen et al., 2011).

Brain functional sources exist at different spatial scales (e.g., model orders), and each spatial scale contains its own functional sources with unique functional information. Most studies have used single model-order ICA. However, functional interactions may occur among functional sources within and between different spatial scales (e.g., large networks interact with small networks). The functional interactions in cross-model orders (between-model orders), may reveal important information and might be ignored if using a single spatial scale to analyze the data (Iraji et al., 2022a, Iraji et al., 2022b). Mutil-model orders (multiple spatial scales) studies, not like traditional single model orders ICA studies (typically with moder order of 20 ∼ 100 components), focuses on combining different model orders (in this study, we combinedly use model order of 25, 50, 75, and 100), to capture the functional information at different ICA dimensionalities as well as the cross-model order information. Our previous research (Meng et al., 2021) has demonstrated that multi-model order classification gave better results compared to single-model order classification, in particular the (typically ignored) cross-model order (cross-spatial scale) information appears to be contributing unique information, which highlights the benefit of multi-model order analysis.

### 2.2 scICA vs classic ‘fully blind’ ICA

Compared to classic ICA, scICA has several advantages, 1) scICA is fully automated ICA, 2) the component ordering and grouping are automatically obtained, and 3) scICA runs at the single subject level. For classic ICA, running on a group can lead to data leakage, running on individual subjects would be prohibitively expensive as it would require sorting and grouping components separately for each subject. Although this process can be done using greedy matching algorithms, it is very messy and error-prone. While ICA followed by regression of the spatial maps onto new data offers one solution, we have previously shown that scICA, which optimizes for independence at the single subject level, outperforms spatio-temporal (dual) regression-based approaches (Salman *et al.,* 2019a). Table 1 shows the comparison scICA and classic ICA.

**Table 1:** Comparison between scICA and classic ICA.

Method	Number of Model orders	Generalized conclusion across different model orders	Reveal observations in between model orders
Classic ICA	Typically focus on one model order	No	No
Multi-order scICA	Combination of different model orders	Yes	Yes

### 2.3 Innovation

In this work, we present an approach that uses multi-model order analysis by leveraging MOO-ICAR to study multi-spatial-scale functional interactions (both within and between spatial scales) in schizophrenia. Multiscale ICA uses multi-model order ICA to estimate brain functional networks at multiple spatial scales (Iraji et al., 2022a, Iraji et al., 2022b). In order to compare our framework, which uses multi-model order analysis by leveraging MOO-ICAR in studying schizophrenia, with other traditional ICA approaches, we made the following tabular summary of the paper's review to give readers a better understanding of the research done in this field (as shown in Table 2).

**Table 2:** Summary of analysis approaches of related work.

	Single scale classic ICA	Multiple spatial scale ICA	Spatially constrained ICA
(Nygård et al. 2012)	Yes		
(Salman et al. 2019) (Deramus et al. 2021)			Yes
(Iraji et al., 2022a, Iraji et al., 2022b) (Meng et al., 2021)		Yes	
Proposed research		Yes	Yes

In summary, the innovation of our work compared to previous work is two-fold, 1) We combined, for the first time, scICA and msFNC, to leverage the benefits of both approaches in order to train a classification model in schizophrenia, and 2) Our framework was built and tested leveraging a large N dataset, and revealed highly replicable group differences and consistent predictive results in detecting group differences in schizophrenia.

## 3 Methods

### 3.1 Dataset and preprocessing

We used two datasets in this study. The first data set, ‘dataset 1′, was used as a discovery and validation dataset, and the second ‘dataset 2′ was used as a replication dataset. Dataset 1 was mainly used to extract predictive features based on which classification was built and validated. Dataset 2 was used to further validate the classification model using the features that were selected from dataset 1.

Dataset 1 was selected from three different studies, one with seven sites (fBIRN: Functional Imaging Biomedical Informatics Research Network), one with three sites (MPRC: Maryland Psychiatric Research Center), and one single site (COBRE: Center for Biomedical Research Excellence). This resulted in a total 827 individuals, including 477 subjects (age: 38.76 ± 13.39, females: 213, males: 264) of typical controls (TC) and 350 schizophrenia individuals (age: 38.70 ± 13.14, females: 96, males: 254). The parameter settings for the resting-state fMRI (rsfMRI) data collected in the fBIRN data were the same across all sites, with a standard gradient echo-planar imaging (EPI) sequence (repetition time (TR)/echo time (TE) = 2000/30 ms, voxel spacing size = 3.4375 × 3.4375 × 4 mm, field of view (FOV) = 220 × 220 mm, and a total of 162 vol). Six of the seven sites used 3-Tesla Siemens Tim Trio scanners, and one site used a 3.0 Tesla General Electric Discovery MR750 scanner. For COBRE data, rsfMRI images were acquired using a standard EPI sequence (TR/TE = 2000/29 ms, voxel spacing size = 3.75 × 3.75 × 4.5 mm, FOV = 240 × 240 mm, and a total of 149 volumes. Data were collected using a 3-Tesla Siemens Tim Trio scanner. The MPRC dataset were acquired using a standard EPI sequence in three sites, including Siemens 3.0 Tesla Siemens Allegra scanner (TR/TE = 2000/27 ms, voxel spacing size = 3.44 × 3.44 × 4 mm, FOV = 220 × 220 mm, and 150 volumes), 3.0 Tesla Siemens Trio scanner (TR/TE = 2210/30 ms, voxel spacing size = 3.44 × 3.44 × 4 mm, FOV = 220 × 220 mm, and 140 volumes), and 3.0 Tesla Siemens Tim Trio scanner (TR/TE = 2000/30 ms, voxel spacing size = 1.72 × 1.72 × 4 mm, FOV = 220 × 220 mm, and 444 volumes). This data has also been used in prior work (Iraji et al., 2021a, Meng et al., 2021).

Dataset 2 contained a total of 815 subjects, collected from several Chinese hospitals, including 326 subjects (age: 29.81 ± 8.68, females: 167, males: 159) of typical controls and 489 SZ individuals (age: 28.98 ± 7.63, females: 229, males: 260). The subjects were Chinese ethnic Han groups. The dataset was recruited from seven sites in China with the same recruitment criterion, including Peking University Sixth Hospital; Beijing Huilongguan Hospital; Xinxiang Hospital Simens; Xinxiang HospitalGE; Xijing Hospital; Renmin Hospital of Wuhan University; Zhumadian Psychiatric Hospital (Yan et al., 2019). The resting-state fMRI data were collected with the following three different types of scanners across the seven sites: 3.0 Tesla Siemens Tim Trio Scanner, 3.0 T Siemens Verio Scanner, and 3.0 T Signa HDx GE Scanner (TR/TE = 2000/30 ms, voxel spacing size = 3 × 3 × 3 mm, FOV = 220 × 220 mm, and 480/360 volumes). Subjects were instructed to relax and lie still in the scanner while remaining calm and awake.

Table 3 displays the summary of the two datasets. The replication dataset (dataset 2) is comprised of individuals from a different ethnic group compared to the discovery dataset (dataset 1). The two datasets were preprocessed according to the same procedures as in our previous study (A. Iraji et al., 2021a). To summarize, preprocessing was mainly performed using the statistical parametric mapping (SPM12, https://www.fil.ion.ucl.ac.uk/spm/) toolbox. First, we discarded the first five volumes for magnetization equilibrium. We then performed rigid body motion correction using the toolbox in SPM to correct subject head motion, followed by the slice-timing correction to account for timing difference in slice acquisition. For each subject, the translation of head motion was less than 3 mm and the rotation of head motion was less than 3° in all axes through the whole scanning process. And the next step, the rsfMRI data of each subject was subsequently warped into standard Montreal Neurological Institute (MNI) space using an echo-planar imaging (EPI) template and smoothed using a Gaussian kernel with a 6 mm full width at half-maximum (FWHM = 6 mm). The voxel time courses were z-scored (variance normalized). To make it consistent, the minimum data length (135 volumes) across all subjects from the two datasets was selected for further analysis.

**Table 3:** Dataset summary.

Dataset	Age	Gender (female/male)	Race	PANSS Score (Sample data)
Dataset 1	TC: 38.76 ± 13.39 SZ: 38.70 ± 13.14	TC: 213/264 (44%) SZ: 96/254 (27%)	No record (from US hospitals)	Total: 143 SZ PANSS total: 29.57 ± 8.53 PANSS positive: 15.35 ± 4.96 PANSS Negative:14.22 ± 5.53
Dataset 2	TC: 29.81 ± 8.68 SZ: 28.98 ± 7.63	TC: 167/159 (51%) SZ: 229/260 (45%)	Chinese ethnic Han (from Chinese hospitals)	Total: 149 SZ PANSS total: 43.76 ± 6.29 PANSS positive: 25.35 ± 4.12 PANSS Negative: 18.41 ± 4.78

### 3.2 Analysis framework

**Fig. 1:**
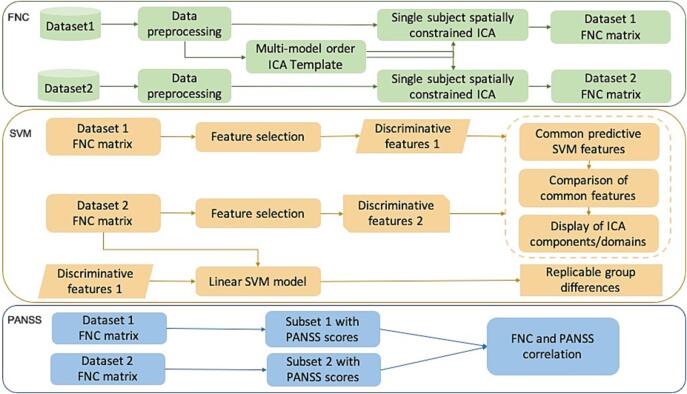
Workflow of our framework. After preprocessing, we calculated the msFNC matrix based on the spatially constrained ICA for both datasets. We then performed feature selection on the msFNC matrix of dataset 1, to find out predictive ICN features. And the next step, we built the SVM model on dataset 2, using the ICN features selected from dataset 1. To find out general predictive ICN features across the different dataset, we repeated the feature selection process on dataset 2, and compared them with the predictive features selected from dataset 1. Thus, we identified consistent predictive features across different datasets. And finally, we extracted a subset of SZ with available symptom scores from dataset 1 and dataset 2, and calculated the linear correlation between msFNC and the symptom score. We then made a comparison between them.

The framework to explore group differences and identify predictability of PANSS scores (Meng et al., 2021) in schizophrenia is provided in Fig. 1. There are three major components in this framework: 1) apply spatially constrained ICA to extract corresponding functional regions and time-courses (TCs), calculate msFNC matrix for each individual; 2) perform feature selection based on the msFNC matrix, and build the SVM classification model; 3) identify predictive ICN domains, and evaluate differences between schizophrenia and control groups.

### 3.3 Spatially constrained ICA analysis

The proposed approach is based on scICA, which incorporates a spatial reference in the ICA algorithm. This allows us to extract only desired ICs, and hence we do not need to run a complete ICA to extract all sources. The scICA analysis was performed using the GIFT software (https://trendscenter.org/software/gift, Calhoun et al., 2001a, Calhoun and Adali, 2012, Iraji et al., 2020, 2021b). We ran scICA on each subject for both dataset 1 and dataset 2, where we utilized ICA with different model orders (25 ∼ 100) to identify ICNs at multiple spatial scales. ICNs were identified from each model order and included components with peak activations in gray matter and low-frequency timecourses (Calhoun et al., 2009). A total of 127 ICNs were hand labeled by experts in our research group from different model orders (15, 28, 36, and 48 from 25, 50, 75, and 100 model orders, respectively). ICNs were grouped into functional domains including cerebellum, cognitive control, default mode, somatomotor, subcortical, temporal, and visual. This template was obtained and labeled using dataset 1 (Iraji et al., 2021a).

### 3.4 msFNC analysis on spatially constrained ICA

msFNC was computed between each pair of ICN time courses by calculating the Pearson correlation coefficient between ICN timecourses (Calhoun et al., 2003a, Calhoun et al., 2003b, Jafri et al., 2008, Allen et al., 2011), which resulted in a 2D symmetric ICN × ICN msFNC matrix for each individual. Each cell of the msFNC matrix represented the functional connectivity between two ICNs. To capture functional interaction across different spatial scales, we calculated the functional network connectivity between each pair of ICNs across all model orders. ICN time courses were interpolated to 2 s for a subset of dataset 1 (15%) with a sampling rate other than 2 s and for all of dataset 2. We aggregated the msFNC matrix from all subjects into an augmented 2D matrix. We then calculate the mean msFNC matrix of all subjects for further analysis.

### 3.5 Feature selection on msFNC

**Fig. 2:**
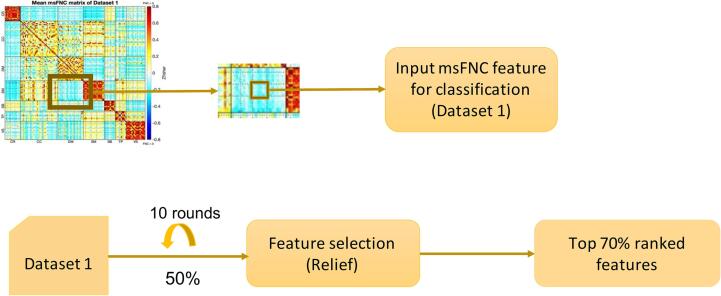
Feature selection workflow. The feature selection process was performed on dataset 1. Each unit in the msFNC matrix was considered a feature. To get a stable result, the selection process was repeated for 10 rounds. We calculated an average weight for each feature across the 10 rounds. We then selected the top 70% predictive features (with higher weights).

The feature selection process was described in Fig. 2. It was performed using only dataset 1. Each msFNC pair was considered as the input feature for classification, and the category of group TC or SZ was considered as the response vector. Given that some of those msFNC features might be non‐informative or redundant for classification, we performed feature selection using Relief (Verma and Salour, 1992) to improve classification performance and speed up computation. Relief calculates a feature score for each feature which can then be applied to rank and select top-scoring features for feature selection. Alternatively, these scores may be applied as feature weights to guide downstream modeling. Relief feature scoring is based on the identification of feature value differences between k nearest neighbor instance pairs. If a feature value difference is observed in a neighboring instance pair with the same class (a 'hit'), the feature score decreases. Alternatively, if a feature value difference is observed in a neighboring instance pair with different class values (a 'miss'), the feature score increases. We set k to 10 in accordance with our previous work (Meng et al., 2021). The function returns the indices of the most important predictors (features) and the weights of the predictors. Feature selection was carried out before classifier training through the recursive feature elimination step. For each round of feature selection, 50% of the training data was selected. We repeated it for ten rounds and retained those features with a high average weight (top 70%) among all the rounds. We thus narrowed the set of features to a subset of the original feature set, eliminating msFNC features' redundancy. The classification model was built based on the selected msFNC feature set.

### 3.6 Support vector machine-based classification (SVM)

The SVM (Verma and Salour, 1992) is a widely used binary classification method due to its ability to deal with high-dimensional data and versatility in modeling diverse sources of data. The SVM has been widely applied in numerous neuroimaging classification studies and has achieved remarkable results due to its excellent generalization performance. Our motivation for using SVM over other approaches was due to its sensitivity, resilience to overfitting, ability to extract and interpret features, and superior performance in fMRI data classification (Martino et al., 2008, Pereira et al., 2009, Ecker et al., 2010, Liu et al., 2013, Vergun et al., 2013, Wang et al., 2019, Saha et al., 2021). To investigate the group differences, we built a binary SVM classifier using a linear kernel (Hsu, 2003), as a straightforward baseline classifier, to demonstrate the practicability of our framework. We also compared the performance of SVM and random forest as a comparison model on a small training set, as SVM outperformed random forest in that case, we utilized SVM as our baseline model.

We built an SVM model on dataset 2, using the ICN features selected from dataset 1 as mentioned in the previous feature selection section. The classification model was trained and cross-validated (10 fold). To obtain stable performance, we iteratively built and evaluated the classification model multiple times on dataset 2. For each iteration, we randomly split the whole dataset 2 into 80% of the training set and 20% of the testing set. The test set was held out for final evaluation. We ran the modeling process for a total number of 100 iterations and evaluated the SVM model based on average specificity, sensitivity, and F1 score (the harmonic mean of the precision and recall) across all iterations.

### 3.7 Compare common predictive features

To identify common predictive ICN features in detecting schizophrenia across different datasets, we repeated the same feature selection procedure on dataset 2, and compared the selected features from dataset 2 with the ones that were selected from dataset 1. As we have a large number of features (in total 8,001 msFNC features, between 127 pairs of ICNs), we wanted to focus on those highly predictive features, and explore a heuristic result. As a result, we selected around 1% (96 features out of 8,001 in total) of top-ranked features from each of the datasets. We then compared the two feature sets and selected the overlapping features. We normalized their feature weights for further comparison. The major goal in this section is to find out consistent predictive ICN features across different datasets, and thus to show the robustness of our study.

### 3.8 Correlation between msFNC and symptom score

We investigated the correlation between msFNC and the symptom scores, measured by the positive and negative syndrome scale (PANSS) (Kay et al., 1987), aiming to evaluate the consistent predictive strength of the msFNC features across datasets from a different view. In theory, the predictive msFNC features should have in general similar correlations with symptom scores across datasets. We extract 143 schizophrenia individuals from dataset 1, and 149 schizophrenia individual subjects from dataset 2 separately, with valid symptom scores (PANSS total, PANSS positive, PANSS negative). We then calculated the linear correlation between the msFNC and the symptom scores for each of the subjects of the two datasets and made a comparison between them.

## 4 Results

### 4.1 msFNC analysis across different databases

**Fig. 3:**
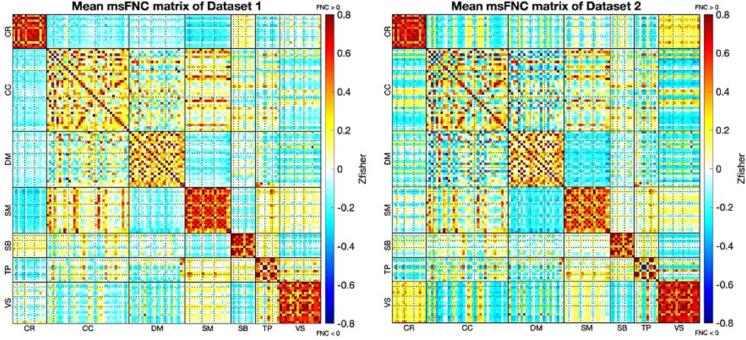
Mean msFNC plot of dataset 1 (left) and dataset 2 (right). We calculated the mean msFNC (z-fisher score) based on the aggregated msFNC matrix of all individuals. The ICNs in these msFNC matrices were sorted by domains first, and within each domain, ICNs were sorted by model orders (from 25 to 100). The dot lines in each domain divide different model orders. ICNs were sorted in the order of cerebellum (CR), cognitive control (CC), default mode (DM), somatomotor (SM), subcortical (SB), temporal (TP), and visual (VS). The overall pattern of the two datasets is similar. Stronger correlations in CR vs. VS and anticorrelations in DM vs. SM, CC vs. DM were seen in dataset 2, and stronger anticorrelations in CR vs. SM in dataset 1.

Fig. 3 presents the mean msFNC (z-fisher score) between 127 ICNs of all model orders, for dataset 1 and dataset 2, separately. The overall patterns of the mean msFNC matrix of dataset 1 and dataset 2 were similar. We observed that the cerebellum (CR), somatomotor (SM), subcortical (SB), temporal (TP), and visual (VS) were highly correlated with themselves in msFNC for both of the datasets, which were more homogeneous than the default mode (DM) and cognitive control (CC) domains.

**Fig. 4:**
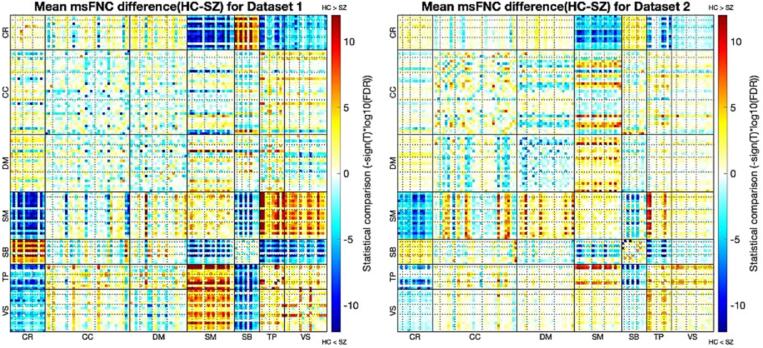
Mean msFNC difference (TC - SZ) matrix for dataset 1 (left) and dataset 2 (right). This figure shows the difference between TC and SZ in msFNC. Blue areas indicate increased msFNC in SZ compared to TC, and red areas indicate decreased msFNC in SZ compared to TC. The intensity values (-sign(T)*log10(FDR)) have been calculated. Increases in msFNC were seen in CR vs. SM, CR vs. VS, CR vs. TP, SB vs. VS, SB vs. TP, SM vs. SB for both datasets. And decreases in msFNC were mainly seen in CR vs. SB, SM vs. VS and SM vs. TP. Discernible group differences were observed in dataset 1, compared to dataset 2, as it shows in the darker regions. (For interpretation of the references to colour in this figure legend, the reader is referred to the web version of this article.)

We then evaluated the group difference between SZ and TC groups for the two datasets. The most dominant increases in msFNC in the SZ group for both datasets were mainly seen between the ICNs of cerebellum vs. somatomotor, cerebellum vs. temporal, and cerebellum vs. visual. Increases in msFNC were also seen between the ICNs of subcortical vs. visual, subcortical vs. temporal, and subcortical vs. somatomotor. In addition, both datasets show a relatively large decrease in the SZ groups compared to the TC groups between the ICNs of the subcortical vs. cerebellum, somatomotor vs. visual, somatomotor vs. temporal. Fig. 4 displays the overall group differences between SZ and TC for the two datasets. Statistical comparison (intensity values (-sign(T)*log10(FDR), T: t-values from two-sample t-tests, FDR: corrected p-values obtained from two-sample t-tests between SZ and TC group) was compared between the two datasets.

**Fig. 5:**
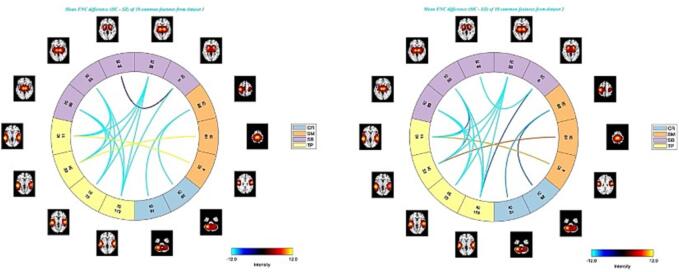
Connectogram of mean msFNC difference (TC - SZ) matrix of 18 common predictive features for dataset 1 (left) and dataset 2 (right). The common features show strong increases in msFNC in the domains of SB vs. TP, SB vs. SB, CR vs. SM, and decreases in SM vs. TP, for both of the datasets.

As we mentioned, we selected around 1% of top-ranked features from each of the datasets and then compared the two feature sets and retained the overlapping features, which resulted in 18 common predictive features. Fig. 5 shows the Connectogram of the average msFNC difference between TC and SZ for the 18 common predictive features between the two datasets. The common predictive features have shown distinct group differences between TC and SZ in msFNC for both datasets. The selected common features were considered the most predictive features in detecting group differences in schizophrenia, as they were selected separately from each dataset by feature selection. These common features identified ICN domains that are highly related to schizophrenia. It is observed that the common predictive features also show consistent group differences and consistent signs (positive vs negative) for the msFNC in the two datasets.

### 4.2 Support vector machine-based classification (SVM)

We evaluated the performance of the SVM model as shown in Table 4. As we mentioned, the SVM model was built using the predictive features selected from dataset 1, and then trained and verified on dataset 2. The performance was averaged across 100 iterations of modeling. As shown in the table, the average accuracy of the classification model was 81.4%, with a precision of 82.6%, 87.6% recall, and 84.9% F1 score.

**Table 4:** The average performance of the SVM model for 100 iterations.

	F1	Precision	Recall	Accuracy
SVM	0.8493	0.8259	0.8759	0.8137

**Fig. 6:**
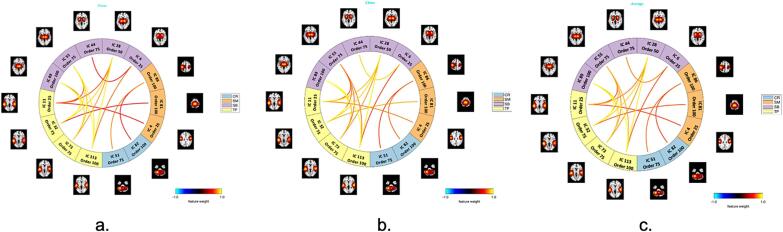
Connectogram of feature weights of the 18 common features, for dataset 1 (a), dataset 2 (b), and the combined dataset (c). The common features have shown strong predictive strength in predicting group differences of TC and SZ, for both of the datasets. Statistically, the relevance level of a relevant feature is expected to be larger than zero and that of an irrelevant one is expected to be zero (or negative). The SB, TP, SM, and CR contribute most to the classification, for both of the datasets. The ICN features in domain TP (yellow) vs. SB (purple) generally have higher weights compared to other ICN domains, which indicates their predictive strength in detecting group differences of TC and SZ. (For interpretation of the references to colour in this figure legend, the reader is referred to the web version of this article.)

To better understand the ICN domains that the 18 common predictive features connected and the features’ contribution in detecting group differences, we show the connectograms (as shown in Fig. 6) of the feature weights of the common features. The majority of the common features fell into the ICNs domains of subcortical vs. temporal, the rest of them fell into the ICNs domains of somatomotor vs. temporal, somatomotor vs. cerebellum, and within the subcortical, which may indicate an important role of these domains in detecting schizophrenia. The lines within the circles represent ICN features, and the outer circles indicate the ICN domains the features belong to. As we mentioned, the common features fell into four ICN domains, cerebellum, somatomotor, subcortical, and temporal, with 2, 3, 5, and 4 ICs in each of them separately. It is observed that the majority ICs (11 out of 14) involved were from higher model orders (75 ∼ 100), and most of them were from between model orders. The subfigure Fig. 5c the averaged feature weights of the common features of the combined two datasets.

### 4.3 Correlation between msFNC and symptom scores

**Fig. 7:**
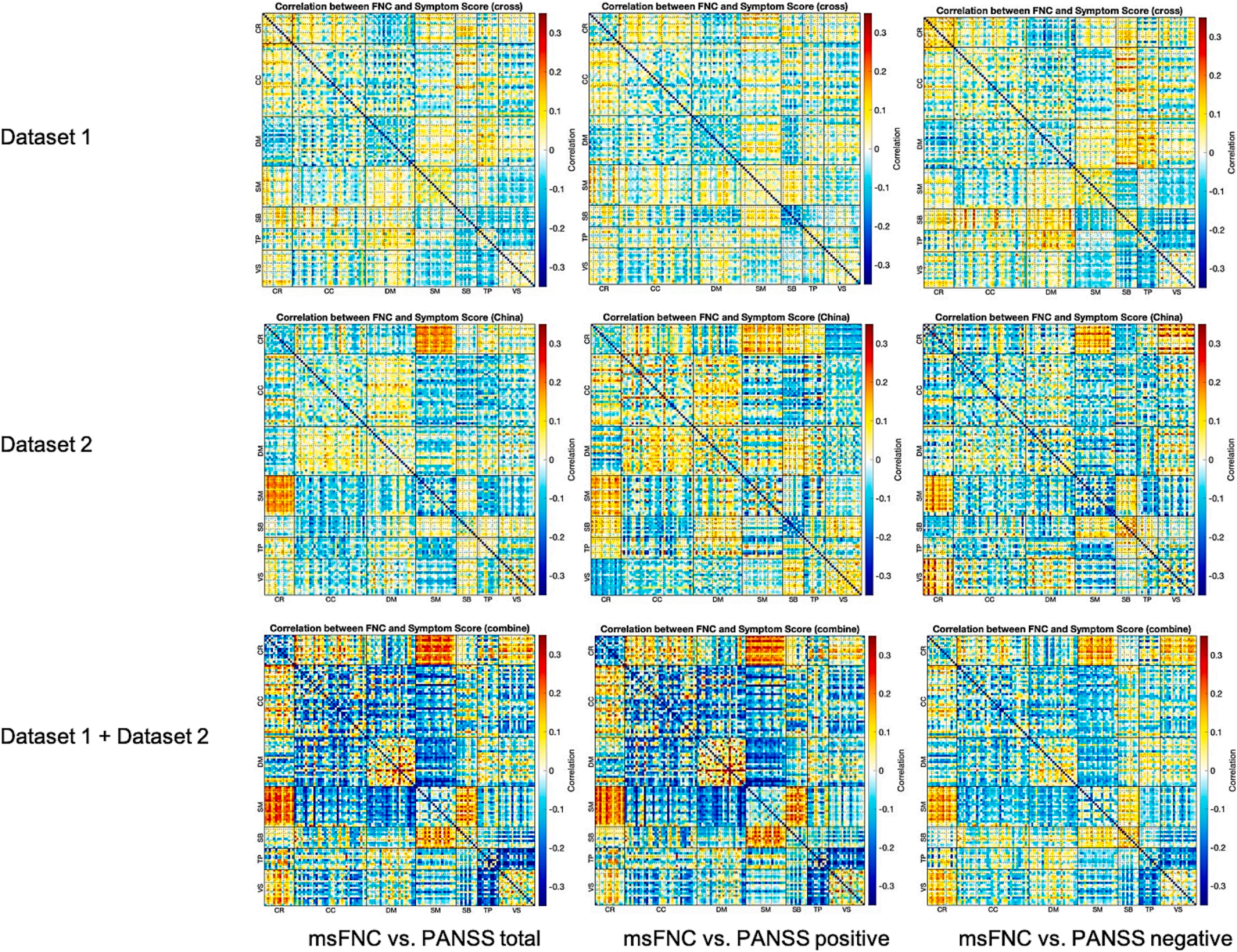
Correlations between msFNC and symptom score, for dataset 1 (the first row), dataset 2 (the second row), and the combined dataset of the two datasets (the third row). Each row shows the correlations between msFNC and the PANSS total (left), PANSS positive (middle) and PANSS negative (right), separately. We extracted 143 and 149 SZ with valid PANSS(total, positive and negative) scores from dataset 1 and dataset 2 separately. We calculated linear correlations between the msFNC matrix and the PANSS scores for each SZ patient. It is noticeable that the correlations between msFNC and the symptom scores in the ICN domain of CR vs. SM are stronger in dataset 2, compared to dataset 1.

In order to detect the correlation between msFNC and symptom scores, we calculated the linear correlation between the msFNC of each subject and its symptom scores (including PANSS total, PANSS positive, and PANSS negative). The following figures show the correlation matrix between msFNC and symptom scores in the two datasets. It is observed that the correlation in the ICN domain of somatomotor vs. cerebellum in dataset 2 was noticeably stronger than that of dataset 1. And also, dataset 2 shows generally stronger anticorrelations between msFNC and symptom scores in the ICN domains of somatomotor vs. visual, somatomotor vs. temporal, and somatomotor vs. cerebellum, compared to dataset 1, which have indicated the major differences of the two datasets. In addition, both of the datasets have shown strong anticorrelations within the subcortical domain between the correlation of msFNC and PANSS positive. We then calculated the correlation between msFNC and symptom scores with the combined dataset of the two datasets, as shown on the third row of Fig. 7 (Appendices).

**Fig. 8:**
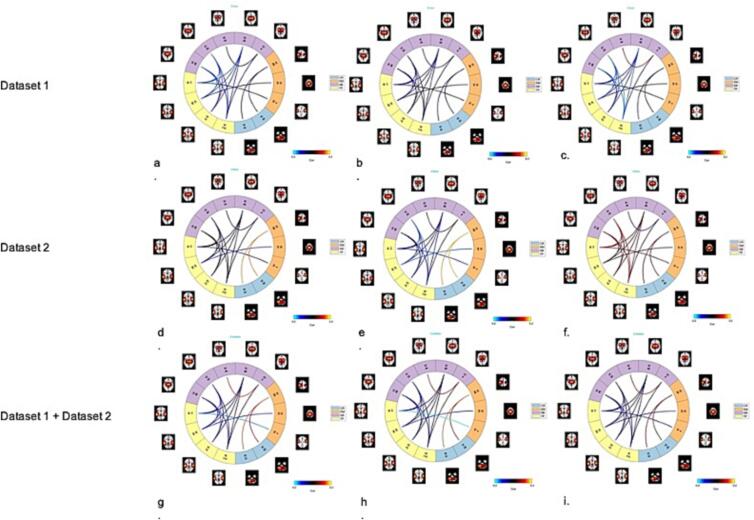
Connectograms of the correlation between msFNC and symptom scores for the 18 common features. The connectograms of the first row and second row show the correlations between msFNC and the PANSS total (a. and d.), PANSS positive (b. and e.) and PANSS negative (c. and f.), for dataset 1 and dataset 2 separately, where PANSS total is the sum of PANSS positive and PANSS negative. The connectograms of the last row are the correlations between msFNC and the PANSS total (g.), PANSS positive (h.) and PANSS negative (i.) for the combined dataset. Linear correlation was calculated between the msFNC matrix and the PANSS scores for each SZ patient. It is seen that the anticorrelations between the ICN domains of TP (yellow) vs. SB (purple) were generally stronger in dataset 1 (the first row) compared to dataset 2 (the second row), as shown in the lighter blue lines within the circles. Stronger correlations between CR (blue) vs. SM (orange) in dataset 2 were also noticeable, as they show in the red lines in the second row, compared to the first row of dataset 1. (For interpretation of the references to colour in this figure legend, the reader is referred to the web version of this article.)

Fig. 8 shows the connectograms of the correlation between msFNC and symptom scores for the 18 common predictive features selected from the two datasets. The common predictive features in dataset 2 (the second row) have shown stronger correlations between msFNC and symptom scores (PANSS total on the left, PANSS positive in the middle, and PANSS negative on the right) in the ICN domain of somatomotor vs. cerebellum, compared to the ones in dataset 1 (the first row). The third row of the figure shows the correlation between msFNC and symptom scores of the common features combined of the two common feature sets.

## 5 Discussion and limitation

In this work, we present a framework to extract subject-specific intrinsic connectivity networks from fMRI data at multi-model order spatially constrained ICA. We built our predictive model based on the ICN features selected from one dataset, and trained the model on a different dataset, using the predictive features selected from the first dataset. We then compared the two independent datasets, regarding their msFNC patterns, predictive ICN features, group differences between typical controls and schizophrenia patients, and also the correlations between msFNC and symptom scores. Results have shown consistent predictive strengths of the four ICN domains of the cerebellum, somatomotor, subcortical, and temporal domains, in detecting schizophrenia. The multiscale ICA template was generated from dataset 1. It was also used as a training set of feature selection for the classification model. Dataset 2 was used as an independent dataset on which the classification model was built and tested. Considering the heterogeneity and differences (age and race) in the two datasets, our approach has demonstrated strength in finding consistent SZ changes regardless of these differences. The performance of the classification model reached up to 85% F1 score, 83% precision, and 88% recall. Results suggest the proposed framework would probably provide similar performance if applied to other datasets with different demographics (race, age, gender, etc.), given the replicable evidence we obtained. Our results demonstrated that MOO-ICAR is capable of obtaining subject-specific ICNs with strong independence, which in the meanwhile reduced the computational cost compared to the standard ICA methods. As we observed, the 18 common predictive features fell into four ICN domains, cerebellum, somatomotor, subcortical, and temporal. And the majority ICs involved were from between model orders, for example, subcortical vs. temporal, somatomotor vs. temporal, and somatomotor vs. cerebellum. The results have shown the importance of studying brain functional connectivity at cross-spatial scales.

Compared to classic single-model order ICA, multi-model order ICA has multiple advantages in that: 1) It allows us to use the cross-model order information, which is not possible for a single model order, since there is no cross-model order information or different spatial scales in that case. As we addressed in Section 2.5, many of the predictive features were cross-model features. Moreover, the cross-model is informative and widely applicable to other areas since it allows us to capture coupling between smaller and larger networks. 2) It allows us to visualize networks or nodes at different model orders. 3) It improves classification accuracy. To compare the predictive strength of the proposed classification models using multi-model order ICA and the standard approach (single-model order ICA), we performed classification modeling using standard analysis using single model order (100 component decomposition). The feature selection and parameter tuning process were exactly the same when training the classification models. The resulting accuracy of single-model order ICA was slightly, but significantly, lower (80% vs 81%) and, importantly, was not able to reveal the informative cross-model order information and allow us to evaluate both larger networks as well as subnodes within networks, along with their interactions, simultaneously. 4) Because this is spatially constrained ICA, the multi-model order subsumes the single model-order approach. 5) Group ICA is not ideal for classification since it does not allow fully independent cross-validation, and single-subject ICA without constraint would require extensive matching and be impractical for a large number of subjects.

There are some limitations of the current study. First, for simplicity, we only selected and evaluated a small set (around the top 1%, which resulted in 96 features out of 8001 in total) of top-ranked common ICN features from the two datasets as our preliminary results. The selected 18 common features showed consistent strength in detecting group differences between TC and SZ groups in schizophrenia. However, it is worth exploring a larger range of scales of the common feature set in future work and evaluating the optimal size of the common predictive feature set to build the classification model. This may improve the performance of the predictive model. In addition, we set k to 10 as the number of nearest neighbors when applying Relief for feature selection in accordance with our previous work (Meng et al., 2021). For future work, it would be informative to explore a range of k values to ensure robustness. And also, when we performed feature selection on dataset 1, again for computational reasons, we selected the top 70% ranked features to train the SVM model. In future work, we are aiming to explore a set range of ranked features and find the optimal percentage as the parameter. Furthermore, we used SVM model as our classification model in this work, and even so it performed quickly well. However, more advanced classification models could also be used, including deep learning models. For example, work by (Niu et al., 2019) suggests that a convolutional neural network (CNN) can further improve the performance of classification in schizophrenia. Future work will focus on applying deep learning models to further improve the performance of classification. In this work, we were performing feature selection on the labeled dataset. In future work, we would explore some unsupervised feature selection methods, such as filter methods (Sánchez-Maroño,et al., 2007), wrapper methods (Yang et al., 2013) or embedded methods (Wang et al., 2015), which can be used for unlabeled data, to find the best set of features to build the predictive models. Moreover, we would like to pursue the predictive accuracy of various subtyping of schizophrenia approaches (Shi et al., 2023) in future work. And lastly, in this work, we evaluated the relationship of the FNC imaging measures to the PANSS subscales, and found some evidence of consistency across the different analyses. However, this is not the full story, as symptomatology is complicated and the PANSS positive/negative/general averages represent just one, albeit widely used, summary of the PANSS assessment. Additional analyses could evaluate the relationship of the imaging result to data-driven factors derived from the full 30 question answers on the PANSS assessment. As there are multiple ways to approach this, we defer a full treatment of this additional analysis to future work.

## 6 Conclusions

We reported a new framework for detecting FNC differences between groups at multiple spatial scales using spatially constrained ICA. To the best of our knowledge, this is the initial research work using spatially constrained ICA at multiple spatial scales to predict group differences in schizophrenia. Importantly, the clinical significance of our study is to make multiscale analysis comparable across datasets and the proposed framework can be directly applied to any new dataset. Meaningful consistent predictive msFNC features were selected in the study. The results showed consistent evidence of the four ICN domains cerebellum, somatomotor, subcortical and temporal, especially in detecting aberrant FNCs in schizophrenia on two independent datasets. These results highlight replicable cross-spatial scale msFNC differences which may inform our understanding of the neural patterns linked to schizophrenia. Future work might focus on further replication and potentially focus on interventional approaches targeting the highlighted domains.

## CRediT authorship contribution statement

**Xing Meng:** Conceptualization, Methodology, Investigation, Writing – review & editing, Formal analysis, Writing – original draft, Visualization. **Armin Iraji:** Conceptualization, Methodology, Investigation, Writing – review & editing, Visualization. **Zening Fu:** Data curation, Formal analysis. **Peter Kochunov:** Data curation, Resources. **Aysenil Belger:** Data curation, Resources. **Judy M. Ford:** Data curation, Resources. **Sara McEwen:** Data curation, Resources. **Daniel H. Mathalon:** Data curation, Resources. **Bryon A. Mueller:** Data curation, Resources. **Godfrey Pearlson:** Data curation, Resources. **Steven G. Potkin:** Data curation, Resources. **Adrian Preda:** Data curation, Resources. **Jessica Turner:** Data curation, Resources. **Theo G.M. van Erp:** Data curation, Resources. **Jing Sui:** Writing – review & editing. **Vince D. Calhoun:** Conceptualization, Methodology, Investigation, Writing – review & editing, Funding acquisition, Project administration, Resources.

## Declaration of Competing Interest

The authors declare that they have no known competing financial interests or personal relationships that could have appeared to influence the work reported in this paper.

## Data Availability

The authors do not have permission to share data.
